# Statewide Molecular Epidemiology of *Mycobacterium tuberculosis* Transmission in a Moderate- to Low-Incidence State: Are Contact Investigations Enough?

**DOI:** 10.3201/eid0811.020261

**Published:** 2002-11

**Authors:** Wendy A. Cronin, Jonathan E. Golub, Monica J. Lathan, Leonard N. Mukasa, Nancy Hooper, Jafar H. Razeq, Nancy G. Baruch, Donna Mulcahy, William H. Benjamin, Laurence S. Magder, G. Thomas Strickland, William R. Bishai

**Affiliations:** * Maryland Department of Health and Mental Hygiene, Baltimore, Maryland, USA; † American Public Health Association, Washington, D.C., USA; ‡ Alabama Department of Public Health, Montgomery, Alabama, USA; § University of Alabama at Birmingham, Birmingham, Alabama, USA; ¶ University of Maryland—Baltimore, Maryland, USA; # The Johns Hopkins University, Baltimore, Maryland, USA

**Keywords:** molecular epidemiology, tuberculosis, recent transmission, DNA fingerprinting

## Abstract

To assess the circumstances of recent transmission of tuberculosis (TB) (progression to active disease <2 years after infection), we obtained DNA fingerprints for 1,172 (99%) of 1,179 *Mycobacterium tuberculosis* isolates collected from Maryland TB patients from 1996 to 2000. We also reviewed medical records and interviewed patients with genetically matching *M. tuberculosis* strains to identify epidemiologic links (cluster investigation). Traditional settings for transmission were defined as households or close relatives and friends; all other settings were considered nontraditional. Of 436 clustered patients, 114 had recently acquired TB. Cluster investigations were significantly more likely than contact investigations to identify patients who recently acquired TB in nontraditional settings (33/42 vs. 23/72, respectively; p<0.001). Transmission from a foreign-born person to a U.S.-born person was rare and occurred mainly in public settings. The time from symptom onset to diagnosis was twice as long for transmitters as for nontransmitters (16.8 vs. 8.5 weeks, respectively; p<0.01). Molecular epidemiologic studies showed that eliminating diagnostic delays can prevent TB transmission in nontraditional settings, which elude contact investigations.

Although tuberculosis (TB) remains a major public health threat worldwide (1), its declining incidence in the United States has led health policy makers to develop plans for disease elimination (less than one patient per million) by 2010 (2). Although targeted screening and treatment of latent TB infection has been recommended for groups at high risk (3), learning more about recent TB transmission will help identify specific program interventions that may prevent infection and disease.

Molecular epidemiology has been used to identify groups most at risk for recent TB transmission in high-incidence urban and rural areas of the United States (4–9), but little data have been available from sites with a low-to-moderate disease incidence. Maryland’s varied culture and geography provide a microcosm for the study of TB transmission in the United States. The population of 5.1 million resides in distinct areas: urban (city of Baltimore), suburban (5 counties), and rural coastal and mountainous areas (18 counties). Baltimore reports high rates of homelessness, HIV infection, and illegal drug use. Foreign immigration to suburban and some rural areas of the state has increased by 53% in the past decade, causing Maryland to rank third in the nation in rate of foreign population growth (10).

In spite of the presence of these groups at high risk of acquiring TB, excellent treatment regimens utilizing directly observed therapy (87% vs. 47% nationally) and four-drug initial therapy (89% vs. 77% nationally) resulted in a 15-year decline in disease incidence (11). Since 1989, the state’s TB incidence has remained lower than the national average (4.9/100,000 vs. 5.6/100,000 population, respectively, in 2001) (11), and Baltimore ranks 26th among 31 major U.S. cities for TB incidence (Centers for Disease Control and Prevention [CDC], unpub. data, 2000).

As part of the CDC-supported National Tuberculosis Genotyping and Surveillance Network, we used DNA fingerprinting of *Mycobacterium tuberculosis* isolates and patient information to conduct a statewide epidemiologic study of culture-positive Maryland TB patients. We quantified the problem of recent TB transmission in the state, characterized circumstances and settings for transmission, and used our findings to review programmatic interventions.

##  Methods

### Collection of Isolates and DNA Fingerprinting

*M. tuberculosis* isolates from all patients with a positive culture reported to the Maryland Department of Health and Mental Hygiene (DHMH) between 1996 and 2000 were retrieved from respective reporting laboratories. Restriction fragment length polymorphism (RFLP) analysis of IS*6110* was performed with the standard method (12). Spoligotyping was performed for all matching strains that had six or more IS*6110* copies by using a commercially available kit, according to the manufacturer’s instructions (Isogen Bioscience BV, Maarssen, the Netherlands) (13). Patients with genetically related *M. tuberculosis* strains were considered clustered. For high-copy (more than six) IS*6110* strains, patients whose isolate patterns matched exactly, or differed by one band, were assigned a single cluster designation (14,15). For low-copy (fewer than six) strains, cluster designations were assigned to patients whose isolates matched exactly by RFLP analysis and spoligotyping.

### Demographic and TB Risk Information

For all culture-positive patients with available DNA fingerprints, we obtained routinely reported demographic and risk factor information (HIV infection, homelessness, incarceration, alcohol abuse and illegal drug use, long-term care residence, and foreign birth) from the state case registry. These data were used to compare patients by estimated time of TB acquisition.

### Cluster Investigation

After obtaining genotype results, we abstracted medical records of the clustered patients to determine whether epidemiologic links existed with other patients in the same cluster. We obtained medical histories and information on workplaces, schools, social settings, known or suspected TB exposures, tuberculin skin test results, and contact investigation records. Locatable clustered patients who had no documented links were interviewed to determine whether an existing relationship had eluded the local health department staff who conducted the contact investigations. We assigned epidemiologic links to patients who were named by another TB patient or were in the same place at the same time as another TB patient, even when they did not name each other. When the date and location of specimen collection and laboratory processing suggested that a clustered patient’s specimen was falsely positive, a pulmonologist reviewed medical records and chest radiographs to determine whether clinical TB was likely (16,17). Researchers used standardized forms to abstract records and interview patients. The study was approved by DHMH and CDC’s institutional review boards, and patients signed informed consent forms before interviews.

### Estimated Time of TB Acquisition

Patients with “recent TB” were defined as those who had become infected within 2 years of disease diagnosis by an identified source patient with a matching fingerprint and whose transmission setting was known. Symptom onset had to occur at least 1 month after the onset date of the source’s symptoms. The date was obtained from the patient’s report or conservatively estimated to be 14 days before the date the first positive specimen was collected or the date that treatment was begun, whichever came first. Patients with “probable recent TB” were defined as all clustered patients who had no known transmission from source patients or evidence of past infection, e.g., no history of previous disease or documented positive results of a tuberculin skin test.

The category of “reactivated TB” from latent TB infection was assigned to clustered patients with documented past infection or disease and no identified source case, and to all patients with unique *M. tuberculosis* strain patterns (4,5). Although disease acquisition from a patient residing in another state or from exogenous reinfection could not be completely excluded, we assumed that these events were rare (18,19).

### Traditional and Nontraditional Transmission Settings

 Traditional settings for transmission were defined as those settings routinely investigated during contact investigations, e.g., households and transmission between close friends and relatives in any location. All other settings where transmission occurred were considered nontraditional.

### Time from Symptom Onset to Treatment Initiation

Using only clustered patients as a convenience sample, we compared the times from reported symptom onset to treatment initiation between transmitters (persons who were the source of infection for a patient with recent TB) and nontransmitters (persons who were never identified as a source for another patient). The possibility of transmission was evaluated through July 2002, 19 months after the last patient in the study was reported.

### Exclusions

 Patients with *M. bovis* infection were excluded. Those with a DNA-confirmed TB relapse (disease occurring >12 months after treatment was completed, due to an identical *M. tuberculosis* strain) (20) were counted only for the first disease episode. All patients whose time of TB acquisition was undetermined were excluded, including those whose cultures were negative for *M. tuberculosis* and the first patient in a cluster if no source patient was identified (5). Although the infections of patients >5 years of age were recent by definition, children whose cultures were negative were not included in this molecular epidemiologic study, and those results are described elsewhere (21). Finally, because spoligotyping poorly differentiates clustered *M. tuberculosis* strains with low copy IS*6110* in population-based studies (22), we could not confidently determine when TB was acquired by patients who had low-copy IS*6110* strains and no known source acquired TB. These patients were excluded from our comparison between patients by time of TB acquisition.

### Analysis

Chi-square tests were conducted for all categorical analyses; Fisher exact test was used when expected cell values were <5. Student *t* test was used for continuous variables.

## Results

### Culture-Positive TB Patients

Of 1,554 TB patients reported from 1996 through 2000, a total of 1,198 (77%) had positive cultures. The cluster investigations revealed that specimens from 11 patients were false positive, and these were deleted from our state TB registry. Five patients with *My. bovis* non–BCG were excluded, and three patients who had DNA-confirmed disease relapse were counted once. No instances of exogenous reinfection from a different *M. tuberculosis* strain were identified. DNA fingerprints were available for 1,172 (> 99%) of 1,179 patient isolates.

Of the 1,172 patient isolates, 436 (37%) were grouped in 111 clusters (median patients per cluster 2; mean 3; range 2–19). Eighty-eight (79%) clusters included persons who resided in one or two adjacent jurisdictions within the state. Overall, 155 (36%) clustered patients were epidemiologically linked to another patient in the cluster; among 336 with high-copy IS*6110* strains, 148 (44%) were linked.

### Time of TB Acquisition

 The time of TB acquisition could not be determined for 42 patients who were the first symptomatic patients in their respective cluster and had no known source patient, and 145 patients who had low-copy IS*6110* strains and no known source patients (Table 1). These 187 were excluded from our comparison between patients by time of acquisition. However, 29 of the 187 patients were the source for another patient and were included in our analyses of paired source and secondary patients, and of transmitters and nontransmitters.

**Table 1:** Estimated time of infection and disease acquisition among *Mycobacterium tuberculosis* culture-positive patients by DNA cluster status^a^

DNA cluster status of patients’ isolates	No. patients with recent TB	No. patients with probable recent TB	No. patients with reactivated TB	No. patients with unknown time of TB acquisition	Totals
Clustered strains with >6 IS*6110* copies	89	82	56^b^	42^c^	269
Clustered strains with <6 IS*6110* copies	22	0	0	145	167
Nonclustered strains	4^d^	0	732	0	736
Total	115	82	788	187	1,172

^a^TB, tuberculosis. ^b^History of previous positive tuberculin skin test or extensive past exposure to a patient. ^c^First patient in a cluster by estimated date of symptom onset and no identified source patient. ^d^Known link to another patient outside the study area or timeframe whose isolate had the same DNA fingerprint.

Of the 985 patients with a known time of infection and subsequent disease, 115 (12%) had recent TB and an additional 82 (8%) had probable recent TB. Fourteen (17%) of these 82 had documented previous negative skin tests. Our extensive case review showed no sources for 56 clustered patients who had documented histories of past infection or disease. We presumed that these 56 patients plus the 732 patients with unique *M. tuberculosis* strains had reactivated disease (n=788).

Patients with recent TB were significantly more likely than patients with probable recent TB to be young and U.S. born, but the proportions of patients with urban residence, HIV infection, illegal drug use, and homelessness were similar for both groups (Table 2). Among the 25 patients with probable recent TB who were >64 years old, 4 were foreign-born, 10 were users of illegal drugs, and 2 were homeless. Patients with recent TB were more likely than those with reactivated disease to be urban residents, young, black, U.S.-born, homeless, HIV-infected, and abusers of alcohol or illegal drugs.

**Table 2:** Selected characteristics of culture-positive patients with comparison between categories

	No. patients with recent TB (%) (N=115)	No. patients with probable recent TB (%) (n=82)	No. patients with reactivated TB (%) (n=788)	p value	p value
Characteristic	Group A	Group B	Group C	A vs. B	A vs. C
Residence					
Baltimore City	57 (50.0)	38 (46.3)	157 (19.9)	0.66	<0.001
Other state jurisdictions	58 (50.0)	44 (53.7)	631 (80.1)		
					
Age group (yrs)					
0–14^a^	6 (5.2)	3 (3.7)	5 (0.6)	<0.001	<0.001
15–24	21 (18.3)	7 (8.5)	86 (10.9)		
25–44	46 (40.0)	27 (32.9)	275 (34.9)		
45–64	33 (28.7)	20 (24.3)	178 (22.6)		
>65	9 (7.8)	25 (30.4)	244 (31.0)		
					
Race/ethnicity					
White, non-Hispanic	20 (17.4)	17 (20.7)	162 (20.6)	0.03	<0.001
Black, non-Hispanic	89 (77.4)	51 (62.2)	341 (43.3)		
Hispanic	1 (0.9)	5 (6.1)	84 (10.7)		
Asian	5 (4.3)	9 (11.0)	200 (25.4)		
Native American	0	0	1 (0.1)		
					
Country of birth					
United States	105 (91.3)	66 (80.5)	360 (45.7)	0.03	<0.001
Other	10 (8.7)	16 (19.5)	428 (54.3)		
					
Long-term care resident					
Yes	7 (6.1)	5 (3.3)	25 (3.2)	1.00	0.11
No	108 (93.9)	77 (96.7)	763 (96.8)		
					
Homeless					
Yes	18 (15.7)	8 (9.8)	23 (2.9)	0.22	<0.001
No	97 (84.3)	74 (90.2)	765 (97.1)		
					
Prison resident					
Yes	13 (11.3)	10 (13.1)	21 (2.7)	0.85	<0.001
No	102 (88.7)	72 (86.9)	767 (97.3)		
					
Uses illegal drugs or abuses alcohol					
Yes	53 (46.0)	30 (36.6)	76 (9.6)	0.18	<0.001
No	62 (54.0)	58 (63.4)	712 (90.4)		
					
HIV-infected					
Yes	28 (24.3)	19 (23.2)	75 (9.5)	0.85	<0.001
No	87 (75.7)	63 (76.8)	713 (90.5)		
^a^Includes one child < 6 years old without a known source patient; the case was classified as recent based on age.					

### Risk Factors among Paired Source and Secondary Patients

Of the 115 patients with recent TB, 114 had 69 sources with available risk information. The mean number of secondary patients per source was 1.6 (median 1; range 1–12). Six (5%) of the 114 secondary patients acquired a resistant *M. tuberculosis* strain (primary resistance) from their source; 2 of these 6 were foreign-born. Five patient-strains were resistant to streptomycin and one was resistant to isoniazid. Risks, particularly illegal drug use, were frequently the same for respective source and secondary patients. Risks were identical (e.g., both source and secondary patients were injection drug users, homeless, HIV-infected) for 47 (72%) of 65 patient pairs aged 15–44 years. We found no transmission from U.S.-born persons to foreign-born persons. Other than birth in a country with a high disease incidence, only 2 (11%) of 18 foreign-born sources had risks compared with 46 (90%) of 51 U.S.-born sources (p<0.001). Foreign-born persons were the sources for all 10 foreign-born secondary patients and eight U.S.-born secondary patients. Among the latter, two were young children who acquired infection from a relative. Nonhousehold transmission from foreign-born persons to the remaining six U.S.-born persons occurred in a school, a hospital (22), two churches, and two workplaces. Five of these U.S.-born patients were immunocompetent, and their only risk for TB was exposure to the infectious source patient.

### Identification of Recent Transmission before and after Genotyping

Source cases and settings of transmission were identified for all instances of recent transmission except one, a 3-year-old child (n=114). Fifty-six (49%) patients with recent TB acquired their infection and disease in nontraditional settings (Table 3). Almost two-thirds of the recent patients’ epidemiologic links to their source patients were identified by routine contact investigations before genotyping. Patients identified by contact investigations were significantly less likely to have acquired TB in nontraditional settings than those identified by cluster investigations (23/72 vs. 33/42, respectively; p<0.001).

**Table 3:** Identified transmission settings for 114 patients with recently acquired tuberculosis (TB)

Settings	Total patients with known settings (%)	Setting identified by routine contact investigation (%)	Setting identified by DNA cluster investigation (%)
Traditional Household Close relative Close friend Nontraditional Hospital (23,28) Other workplace (24) Social club (25) Homeless shelter Bar Prison/jail (26) Store (27) Church Nursing home School Ship Mortuary (29) Total	28 (24.6) 13 (11.4) 17 (14.9) 10 (8.8) 6 (5.3) 11 (9.6) 5 (4.4) 10 (8.8) 5 (4.4) 2 (1.8) 2 (1.8) 1 (0.9) 1 (0.9) 1 (0.9) 114 (100.0)	25 (34.7) 13 (18.1) 11 (22.2) 5 (6.9) 6 (8.3) 7 (9.7) 0 1 (1.4) 3 (4.2) 0 0 0 0 1 (1.8) 0 (1.4) 72 (100.0)	3 (7.1) 0 6 (14.3) 5 (11.9) 0 4 (9.5) 5 (11.9) 9 (21.4) 2 (4.8) 2 (4.8) 2 (4.8) 2 4.8) 1 (2.4) 0 (2.4) 42 (100.0)

The importance of nontraditional settings among persons at high risk was influenced in part by large outbreaks (three or more secondary patients) (23–29). Nine of these began in nontraditional settings and ultimately expanded to traditional settings, and cluster investigation identified additional outbreak-related infections in patients who had not been identified through routine contact investigations (24–26).

TB acquisition in nontraditional settings was associated with age >14 years (p=0.033, when differences between older and younger patients were compared), U.S. birth (p=0.012, compared to foreign birth), and illegal drug use (p<0.001, comparing differences between users and nonusers). At least 5 of 15 patients who acquired TB in public settings, i.e., churches, hospitals, a school, and a store, had only brief or distant (casual) exposure to a highly infectious person (27,28). Nine (60%) of the 15 had no apparent TB risk factor except exposure to their source patient.

TB transmission occurred in households for all 10 foreign-born persons with recent TB, and the sources for all but one foreign-born patient were found by contact investigations (Table 4). Cluster investigations were significantly more likely than contact investigations to identify source cases for patients who were homeless, abusers of alcohol, or both. Recent patients with other common TB risk factors, i.e., HIV infection, illegal drug use, incarceration, and long-term care residence, were equally likely to have epidemiologic links identified by cluster or contact investigations.

**Table 4:** Comparison of selected risk-group characteristics of 114 recent tuberculosis (TB) patients by method of source patient identification

Characteristic	Total recent TB patients (n=114) (%)	Source patient identified by routine contact investigation (n=72) (%)	Source patient identified by cluster investigation (n=42) (%)	p value
Residence				
Baltimore city	56 (49.0)	33 (45.8)	23 (54.8)	0.38
Other state jurisdictions	58 (51.0)	39 (54.2)	19 (45.2)	
				
Country of birth				
United States	104 (91.3)	63 (87.5)	41 (97.6)	0.07
Other	10 (8.7)	9 (12.5)	1 (2.4)	
				
Long-term care resident				
Yes	7 (6.1)	3 (4.2)	4 (9.5)	0.25
No	107 (93.9)	69 (95.8)	38 (90.5)	
				
Homeless				
Yes	18 (15.8)	6 (8.3)	12 (28.6)	0.004
No	96 (84.2)	66 (91.7)	30 (71.4)	
				
Prison resident				
Yes	13 (11.4)	8 (11.1)	5 (11.9)	0.86
No	101 (88.6)	64 (88.9)	37 (88.1)	
				
Abuses alcohol				
Yes	40 (35.0)	19 (26.4)	21 (50.0)	0.01
No	74 (65.0)	53 (73.6)	21 (50.0)	
				
Uses injection drugs				
Yes	17 (14.9)	11 (15.3)	6 (14.3)	0.89
No	97 (85.1)	61 (84.7)	36 (85.7)	
				
Uses noninjection drugs				
Yes	35 (30.7)	23 (31.9)	12 (28.6)	0.71
No	79 (69.3)	49 (68.1)	30 (71.4)	
				
HIV-infected				
Yes	28 (24.6)	16 (22.2)	12 (28.6)	0.45
No	86 (75.4)	56 (77.8)	30 (71.4)	

### Time from Symptom Onset to Treatment Initiation

The estimated time of symptom onset was available for 69 transmitters and 99 nontransmitters. TB transmitters were significantly more likely than nontransmitters to have pulmonary disease (68/69 vs. 73/99; p<0.001). Among patients with pulmonary disease, transmitters were more likely than nontransmitters to have lung cavitation (40/68 vs. 14/73; p<0.001) and sputum smears positive for acid-fast bacilli (64/68 vs. 59/73; p=0.034). Among transmitters, the mean time from symptom onset to treatment initiation was 16.8 weeks compared with 8.5 weeks among nontransmitters (median 11 vs. 6 weeks, respectively; p=0.008). Transmitters also were more likely than nontransmitters to have at least one risk factor for TB, e.g., homelessness, HIV infection, alcohol abuse, or illegal drug use, residence in a long-term care facility, incarceration, foreign birth (60/69 vs. 38/99, respectively; p<0.001).

## Discussion

Our 5-year molecular epidemiologic study featured a complete sampling of patients’ isolates from the entire state (30,31) and a multifaceted study site. We also compared our patient groups by time of disease acquisition to more clearly define the relationship between clustering and recent transmission in the state. Even though Maryland has low-to-moderate TB incidence, results from our comparison between groups with recent, probable recent, and reactivated TB were similar to those from studies conducted among clustered and nonclustered patients in high incidence urban and rural areas. Recent and probable recent TB acquisition was associated with patients who were young, homeless, users of alcohol and illegal drugs, HIV-infected, and incarcerated. These findings further support the assumption that clustering is a reasonable, though not exact, surrogate for recent transmission (4–9).

The importance of clustered patients who do not have identifiable links has remained unclear (32). By assuming that clustered patients without links and with histories of old infections or previous TB had reactivated disease, we attempted to be more specific in identifying those for whom recent TB was plausible. Our patients with probable recent TB were older and more likely to be foreign born than were patients with recent TB. Half of the elderly patients had other high-risk factors that made exposure and recent infection likely. Among the foreign-born, acquisition of endemic strains in their countries of origin could account for some clustering (33). However, patients with probable recent TB had risk factors similar to those of patients with known recent TB. The most likely explanation for most clustered patients in this group is that existing epidemiologic links remained unidentified by contact or cluster investigations, and that some had casual exposures to their source patients in unidentified settings.

Patients with reactivated disease were rarely misclassified. Among clustered patients with histories of old infection, disease, or both, our extensive review revealed no source patients. In addition, as of July 2002, we found no instances of exogenous reinfection by a different *M. tuberculosis* strain even among HIV-infected patients. Because genotyping was not conducted in adjoining states, we could not eliminate the possibility of cross-jurisdictional transmission to patients who had unique *M. tuberculosis* strains. Recent TB was transmitted from three patients in Washington, D.C., to four Maryland residents (DHMH, unpub. data, 2001); disease incidence is greater in Washington, D.C., than in Maryland (14.9 vs. 5.3 per 100,000 population, respectively, in 2000) (34). Only 13% of TB patients resided in rural counties that form most of Maryland’s border. With low incidence in adjacent Delaware, Pennsylvania, Virginia, and West Virginia (3.6, 3.1, 4.1, and 1.8 patients per 100,000 population, respectively, in 2000) (34), transmission between states was probably minimal.

### Transmission to and from Foreign-Born Persons

Most recent transmission occurred between U.S.-born persons who had at least one common urban risk factor such as HIV infection or illegal drug use. In contrast, transmission between foreign-born persons occurred exclusively in households among persons who had no other risk except their arrival from a high-incidence country of origin or close exposure to their source patient. We found no instances of transmission between U.S.-born and foreign-born persons. These results differed from other studies, which reported that foreign-born patients who acquired TB from U.S.-born sources shared risks such as homelessness, HIV infection, and illegal drug use with those source patients (35,36). In the past decade, few immigrants and refugees settled in the city of Baltimore where urban risks are common (10). From 1996 through 2000, only 36 (9%) of 423 Baltimore patients were foreign-born compared with 642 (57%) of 1,120 patients in other Maryland areas (DHMH, unpub. data, 2001). In general, foreign migration to Maryland is relatively new (10), and we may observe more shared risks among U.S.- and foreign-born patients as time of residence increases. This study is unique in reporting that infectious foreign-born sources to U.S.-born persons primarily transmitted the disease to persons whose only risk was exposure in their workplace or a public setting, such as a church or school.

### Identification of Recent Transmission before and after Genotyping

In spite of a recommended concentric circle approach for routine contact investigations that includes leisure and social locations (37), we found that investigations usually had been conducted in the homes of patients and rarely extended beyond friends and relatives. Nonetheless, the high proportion of recent patients who acquired TB in these traditional settings clearly represented numerous missed opportunities for disease prevention. Although recent TB was diagnosed among some patients during the initial contact investigation, not all identified contacts had received postexposure tuberculin skin tests or treatment for latent infection (24). More timely and complete contact investigations could reduce the risk for transmission in traditional settings.

Perhaps more importantly, almost half of recent TB cases were acquired in nontraditional settings. Many of these patients were from marginalized groups at high risk, who may have been reluctant or unable to provide names of their associates to contact investigators. However, cluster and contact investigations were equally effective in identifying sources for patients with recent TB who were users of illegal drug users, incarcerated, and HIV-infected, and more aggressive contact investigations could probably not substantially improve patient reporting. Instead, our data suggest that the setting and not the risk group eludes routine contact investigators.

In addition, TB genotyping and cluster investigations indicated unsuspected transmission to immunocompetent persons in public locations such as churches, hospitals, and stores. In these instances, the possibility of casual transmission must be considered. Casual transmission was likely in the store outbreak (27) and conceivably could account for some patients with probable recent TB for whom epidemiologic links were not found. Rarely reported, casual transmission occurs when the bacterial load of the source patient is high, the infecting organism has inherent increased virulence, or the environment is enclosed (27,38). Without creative intervention, the proportional contribution of casual transmission will increase substantially as the disease incidence approaches elimination.

### Delayed Diagnosis Among Transmitters

The mean time between reported symptom onset and initiation of treatment among transmitters was twice that identified for nontransmitters. Whether treatment delays are due to patients who delay in seeking care or to providers who do not include TB in the differential diagnosis, treatment delays provide ample time for pulmonary TB patients to develop smear-positive disease and cavitation (39–41). Our findings led to a study to determine what time period defines a diagnostic delay and to identify related client and provider factors that will guide future program interventions (42).

## Conclusion

Even with excellent treatment indices, one sixth of Maryland’s patients with positive cultures had recent or probable recent disease. The new guidelines for targeted testing and treatment for latent TB infection (3) will require time and substantial resources for successful implementation, and more practical and timely interventions are needed to minimize TB transmission. In the figure, we summarize the program activities that are needed to reduce transmission from infectious TB patients in the various scenarios described in this article. Program implications include the need for improved contact investigations tailored more carefully to each patient’s particular situation, with increased emphasis on activities and patient contacts outside the immediate household. However, contact investigations cannot fully address the problem of transmission in nontraditional settings. Decreasing diagnostic delays can potentially eliminate large point source clusters and substantially reduce transmission in both traditional and nontraditional settings. This method may be the only way to prevent casual transmission. Additional molecular epidemiologic investigations are needed to determine the importance of casual transmission, clarify the importance of clustered patients without links to other patients, and evaluate the long-term effectiveness of new program interventions.

**Figure:**
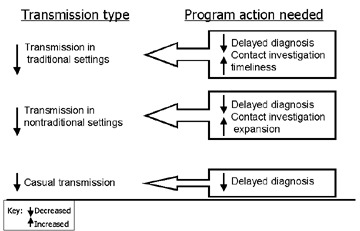
Actions needed to decrease recent tuberculosis transmission in various settings. Decreasing diagnostic delays can potentially eliminate large point source clusters and substantially reduce transmission in both traditional and nontraditional settings. CI, contact investigation.
